# Evaluation of the SARS-CoV-2-IgG response in outpatients by five commercial immunoassays

**DOI:** 10.3205/id000066

**Published:** 2020-09-16

**Authors:** Nele Wellinghausen, Meike Voss, Ralitsa Ivanova, Susanne Deininger

**Affiliations:** 1 MVZ Labor Ravensburg, Ravensburg, Germany

**Keywords:** SARS-CoV-2-IgG, serology, antibody response, COVID-19, immunoassay

## Abstract

Commercially available immunoassays have been developed for sensitive and specific detection of antibodies against SARS-CoV-2. While high sensitivity has been reported in hospitalized COVID-19 patients, little is known about the performance of the assays in ambulatory patients. Therefore, we evaluated the SARS-CoV-2-IgG response in 51 SASR-CoV-2-PCR-confirmed outpatients with five commercial immunoassays. The sensitivity in serum samples, collected at a median of 24 days after onset of symptoms, detected by the Anti-SARS-CoV-2-ELISA IgG (Euroimmun), EDI™ Novel Coronavirus COVID-19 IgG ELISA (Epitope Diagnostics), Liaison^®^ SARS-CoV-2 S1/S2 IgG (Diasorin), SARS-CoV-2 IgG on the Architect™ i2000 (Abbott), and Elecsys^®^ Anti-SARS-CoV-2 (IgM/IgA/IgG) on the cobas™ e801 (Roche) was 84.3%, 78.4%, 74.5%, 86.3%, and 88.2%, respectively. The sensitivity in serum samples, collected >20 days after onset of symptoms, varied between 75.0% and 90.0%, and in samples, collected at least 28 days after onset of symptoms, did not increase, except in the Anti-SARS-CoV-2-ELISA IgG by Euroimmun (90.0%). There was not an obvious association between the type of the antigen (N versus S protein) and the overall sensitivity of the assays. Our results show significant individual differences of the IgG response against SARS-CoV-2, additionally confirmed in three patients with follow-up serum samples and seven asymptomatic but PCR-positive contact persons. In conclusion, our study shows that commercially available immunoassays detect SARS-CoV-2-IgG or total antibodies in outpatients with a satisfying sensitivity, but lower than that reported for hospitalized patients. In asymptomatic persons the SARS-CoV-2-IgG response may even be absent in a relevant percentage of persons.

## Introduction

The severe acute respiratory syndrome coronavirus 2 (SARS-CoV-2) is a new coronavirus which causes an acute respiratory disease, named COVID-19. It emerged in China in December 2019 and led to a worldwide pandemic, declared by the World Health Organization (WHO) on March 11^th^ 2020. As of August 25^th^, more than 23 million cases have been recorded worldwide. 

While diagnosis of acute infection with SARS-CoV-2 is done by real-time polymerase chain reaction (RT-PCR) in respiratory samples there is still an increasing demand on serological testing for both epidemiological studies and the assessment of infection status in individuals. Recent studies have confirmed the suitability of various commercial immunoassays including high-throughput random access assays for the determination of SARS-CoV-2-IgG in COVID-19 patients [1], [2], [3], [4]. Available assays detect antibodies either against the spike (S) protein or against the nucleocapsid (N) protein. Antibodies against the N protein are mounted early in disease while the S protein has been shown to be the target for neutralizing antibodies [5], [6]. 

SARS-CoV-2-IgG were detected second to third weeks after onset of symptoms in up to 100% of hospitalized patients by use of various commercial immunoassays [2], [7], [8], [9], [10], [11], [12]. It has been shown that SARS-CoV-2-IgG titers were higher in critical cases compared to less critical patients and that severe ill patients seroconverted earlier than mild cases [7], [13], [14]. Therefore, it might be assumed that the serological response in outpatients with a less severe clinical status differs from that of hospitalized patients. Outpatients, mildly infected or even asymptomatic contact persons are, however, the main target population for a serological screening in order to evaluate the disease epidemiology. Consequently, this group represents the vast majority of patients, requesting SARS-CoV-2-IgG testing in our laboratory. So, we evaluated the SARS-CoV-2-IgG response (and SARS-CoV-2 total antibody response, respectively, in one immunoassay) in 51 outpatients with past SARS-CoV-2 infection confirmed by RT-PCR as well as 7 asymptomatic contact persons with past positive SARS-CoV-2-PCR.

## Materials and methods

### Serum samples

All serum samples were sent to our laboratory for SARS-CoV-2-IgG determination between March 24^th^ and May 6^th^ 2020 from outpatients. All patients had a positive result of SARS-CoV-2-RT-PCR in a nasopharyngeal swab (at least 7 days before serum collection) in our laboratory information system (LIS). Information about clinical symptoms, the day of onset of symptoms and past hospital treatments for COVID-19 was obtained. Altogether, 60 serum samples were obtained from 51 patients, with clinical symptoms and confirmed-PCR, ambulatory treated SARS-COV-2 infection, fulfilling the clinical diagnostic criteria of the Robert-Koch-Institut (https://www.rki.de/). All patients recovered at the time point of the blood collection. In addition, 7 serum samples from 7 asymptomatic persons with a positive SARS-CoV-2-PCR in the past (before 9 to 56 days) were investigated. All these seven persons were contact persons to PCR-confirmed COVID-19 patients. 

### Immunoassays for SARS-CoV-2 antibody testing

The Anti-SARS-CoV-2-ELISA IgG (Euroimmun, Luebeck, Germany, antigen S1 spike protein) and the EDI™ Novel Coronavirus COVID-19 IgG ELISA (Epitope Diagnostics, San Diego (CA), USA, antigen N protein) were performed fully automated on the Euroimmun Workstation ELISA and the DSX processor system (Dynex Technologies, Denkendorf, Germany), according to the manufacturer’s instructions and within three days after receiving of samples. The results of EDI™ ELISA were calculated as a ratio of the optical density (OD) of the serum sample divided by the OD of negative control plus 0.18 according to the manufacturer’s instructions (negative <0.9, equivocal 0.9–1.09, positive ≥1.1). In addition, the following fully automated random access assays were evaluated: Liaison^®^ SARS-CoV-2 S1/S2 IgG (antigen S1 and S2 spike protein) on the Liaison^®^XL (Diasorin, Dietzenbach, Germany), SARS-CoV-2 IgG (antigen N protein) on the Architect™ i2000 (Abbott, Wetzlar, Germany), and Elecsys^®^ Anti-SARS-CoV-2 (antigen N protein, no differentiation of IgM, IgA and IgG) on the cobas™ e801 (Roche Diagnostics, Mannheim, Germany). On the random access platforms, the samples were measured within one day in batches. Samples were stored at 4–8°C for up to one week, then frozen at –20°C and thawed only once.

### SARS-CoV-2 RT-PCR testing

SARS-CoV-2-RNA detection by real-time RT-PCR from nasopharyngeal swabs was performed within routine diagnostics according to the manufacturers’ instructions with the cobas^®^ SARS-CoV-2 assay on the cobas^®^ 6800 analyzer (Roche Diagnostics, target genes envelope (E) gene and open reading frame (orf) 1 region), the AmpliGnost SARS-CoV-2 E-Gen qPCR (Privates Institut für Immunologie und Molekulargenetik (PIIM), Karlsruhe, Germany) with the cobas^®^ omni channel reagent kit (Roche) on the cobas^®^ 6800 analyzer and the AmpliGnost SARS-CoV-2 E-Gen PCR (PIIM) and AmpliGnost SARS-CoV-2 N-Gen PCR (PIIM) on the LightCycler^®^480 II (Roche). 

## Results

### Comparison of sensitivity of five commercial immunoassays in outpatients

A collection of 60 serum samples from 51 PCR-confirmed outpatients was used for the comparison of sensitivity of the five commercial immunoassays. These sera were sampled 10 to 54 days (median 24 days) after onset of symptoms. A calculation of sensitivity was done firstly irrespective of the time point of serum sampling after onset of symptoms. Equivocal results were counted as negative (n=2 in the Anti-SARS-CoV-2-ELISA IgG (Euroimmun) and the Liaison^®^ SARS-CoV-2 S1/S2 IgG, n=4 in the EDI™ IgG ELISA). The sensitivity ranged from 74.5% to 88.2% (Table 1 (Tab. 1)). The highest sensitivity was achieved with the assays by Abbott and Roche followed closely by the Euroimmun. In samples collected >20 days after onset of symptoms, the sensitivity varied between 75.0% and 90.0% and was higher than in samples collected within the first three weeks in all assays apart from the EDI™ IgG ELISA (Table 1 (Tab. 1)). In a subgroup of samples collected at least 28 days after onset of symptoms (n=20) the sensitivity did not increase further apart from the measurement in the Anti-SARS-CoV-2-ELISA IgG (Euroimmun) (Table 1 (Tab. 1)). 

In each of three patients three follow-up serum samples were availalbe. Patients 1 and 2 were a couple with mild illness, anosomia and without fever. Patient 3 had fever and cough, followed by anosmia and dysgeusia. All five assays showed positive results already in the first serum sample in patient 3. Even though there were marked differences between the assays in the time point of seroconversion in the patients 1 and 2 (Table 2 (Tab. 2)). 

Seven serum samples from seven asymptomatic contact persons with PCR-confirmed SARS-CoV-2 infection were available and tested with all five immunoassays in addition to the above samples from outpatients. All samples were negative in the Anti-SARS-CoV-2-ELISA IgG (Euroimmun), one of each sample was positive in the other immunoassays (Table 3 (Tab. 3)). 

## Discussion

The determination of SARS-CoV-2-IgG antibodies is the method of choice for the evaluation of SARS-CoV-2 seroprevalence. Measurements of SARS-CoV-2-IgG by automated immunoassays preferably run on high-throughput platforms allows a rapid investigation of large sample numbers. Although immunoassays cannot determine the neutralizing ability of SARS-CoV-2-IgG, they facilitate an evaluation of seroprevalence. The results of some tests have been shown to correlate positively with the results of neutralization tests [1], [15].

A comparison of five commercial immunoassays in serum samples taken at least ten days after onset of symptoms from 51 PCR-confirmed COVID-19 outpatients revealed an overall sensitivity of the assays from 74.5% to 88.2%. Highest sensitivities were reached by the SARS-CoV-2-IgG test by Abbott, the Elecsys^®^ Anti-SARS-CoV-2 test by Roche (both N-protein-based tests) and the Anti-SARS-CoV-2-ELISA IgG by Euroimmun (S1-protein-based) while the EDI™ Novel Coronavirus COVID-19 IgG ELISA showed a lower sensitivity. The maximal sensitivity was achieved in samples taken after the third week (using the tests by Roche, Abbott and Epitope Diagnostics) or fourth week after onset of symptoms (using the tests by Euroimmun and Diasorin). It has to be noted that the Elecsys^®^ Anti-SARS-CoV-2 test by Roche detects not only IgG antibodies but total antibodies. An early antibody response may, therefore, in part be due to IgM and/or IgA antibodies. In comparison to data obtained in severely ill and hospitalized patients, where sensitivities up to 100% were found, in samples taken two to three weeks after onset of symptoms, sensitivities of all assays were lower in our cohort of outpatients. SARS-CoV-2-IgG titers were higher in severely ill compared to lesscritical patients [7], [13], [14]. Wajnberg et al., who investigated SARS-CoV-2-IgG by an ELISA test in PCR-confirmed outpatients in New York, reported a positivity rate of 82% at a median of 23 days after onset of symptoms [16]. In contrast, Meyer et al. detected SARS-CoV-2-IgG in 97.7% of serum samples in 44 patients from an outpatient clinic in Switzerland by use of the Anti-SARS-CoV-2-ELISA IgG (Euroimmun) [17]. Regarding the different viral antigens used in the tests, there was no obvious association between the type of the antigen (N versus S protein) and the overall sensitivity of the assays. In addition, there was no association between the type of the antigen and the dynamics of IgG response in the three follow-up patients. Our results show significant individual differences of the IgG response against SARS-CoV-2 as reported by others before [1], [6]. 

Actually, the positivity rate determined in the seven asymptomatic but PCR-positive contact persons was much lower (0 to 1 out of 7 positive). Regarding this low rate of seropositivity, there are different possible explanations:

- Infected persons, who do not develop a clinical disease may possibly combat the coronavirus on the mucosa of their upper respiratory tract, preventing a systemic humoral immune response. According to a recent study SARS-CoV-2-S-protein-specific IgA in nasal and tear fluids may play a role in the primary defense of SARS-CoV-2 and has been found in mucosal samples even in seronegative asymptomatic health care workers [13].
- Previous publications have demonstrated that the humoral immune response towards SARS-CoV-2 depends on the duration of viral antigen exposure [18], [19]. Therefore, it may be postulated that the group of asymptomatic contact persons have been exposed to a lower amount of viral antigen.
- The possibility of false positive RT-PCR results has to be taken into account. Contamination of samples can never completely be excluded, but the following reasons make this explanation unlikely: First, samples that were investigated on the cobas^®^ 6800 analyzer were mainly directly put into the analyzer without a prior opening in the laboratory; second, we retested a large collection of swabs with a weak positive result in an E-gene-specific PCR with another different PCR assay and revealed consistent results (data not shown) and third, many swab samples were positive for two SARS-CoV-2 gene targets.

In summary, our study shows that commercial immunoassays detect SARS-CoV-2-IgG or total antibodies in outpatients with a sensitivity of 75% to 88%, which is lower than the reported one for severe ill and hospitalized patients. Regarding the overall sensitivity, the fully automated assays by Abbott and Roche as well as the ELISA by Euroimmun were superior to the other assays. In asymptomatic persons with past SARS-CoV-2 infection the SARS-CoV-2-IgG response may be absent in a relevant percentage of persons. For the detection of a seroconversion in mild cases and asymptomatic patients, serum should be collected not earlier than three weeks after onset of symptoms or approximately four weeks after contact to a COVID-19 patient. 

## Notes

### Competing interests

The authors declare that they have no competing interests.

## Figures and Tables

**Table 1:**
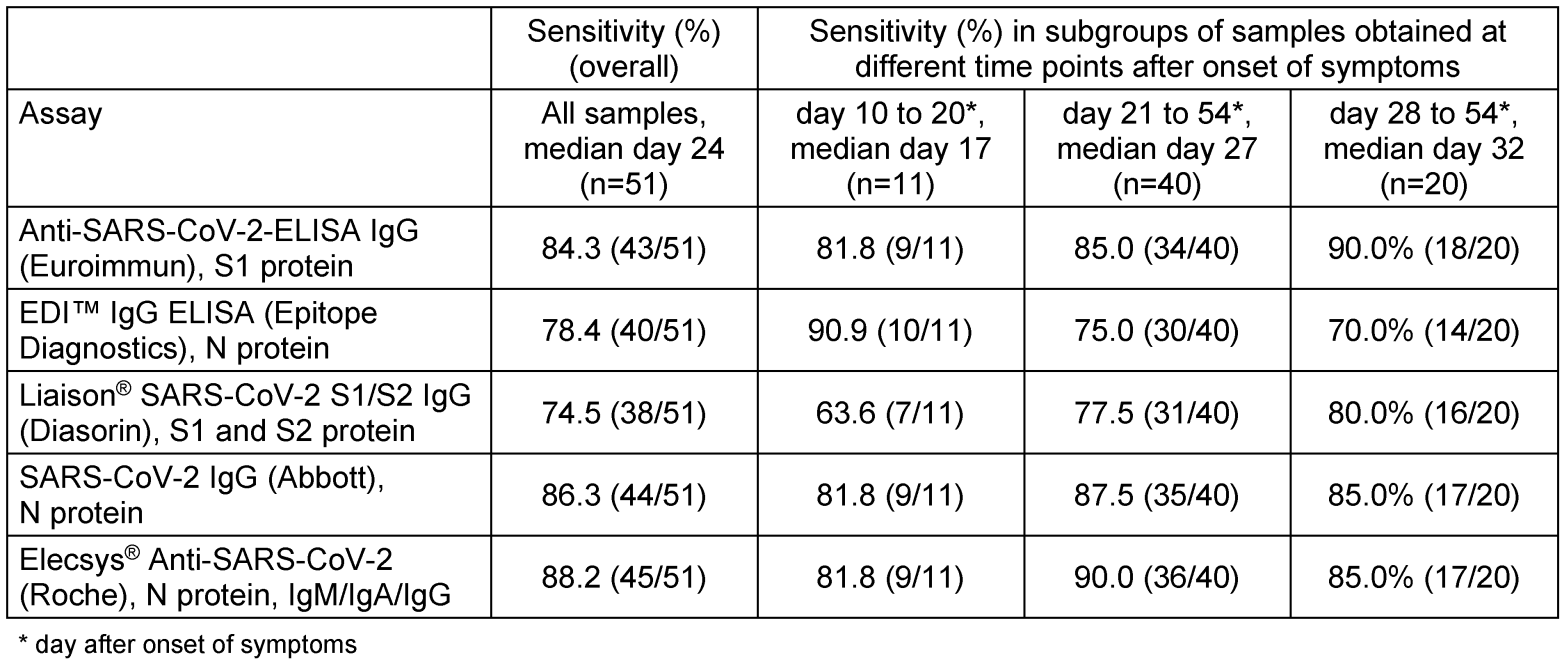
Sensitivity of five commercial SARS-CoV-2 assays in outpatients (n=51)

**Table 2:**
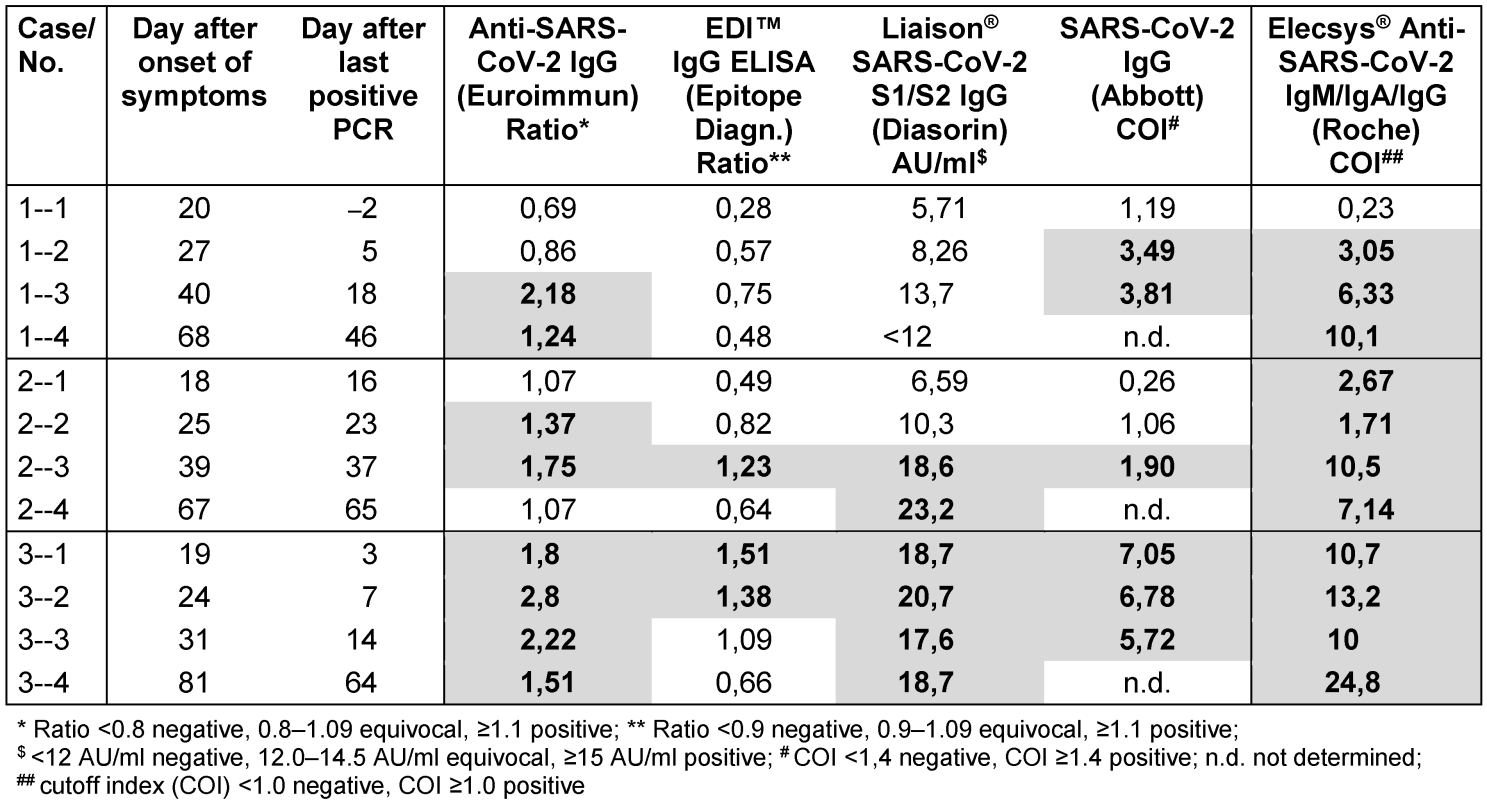
Results of five commercial SARS-CoV-2 assays in follow-up sera from three outpatients

**Table 3:**
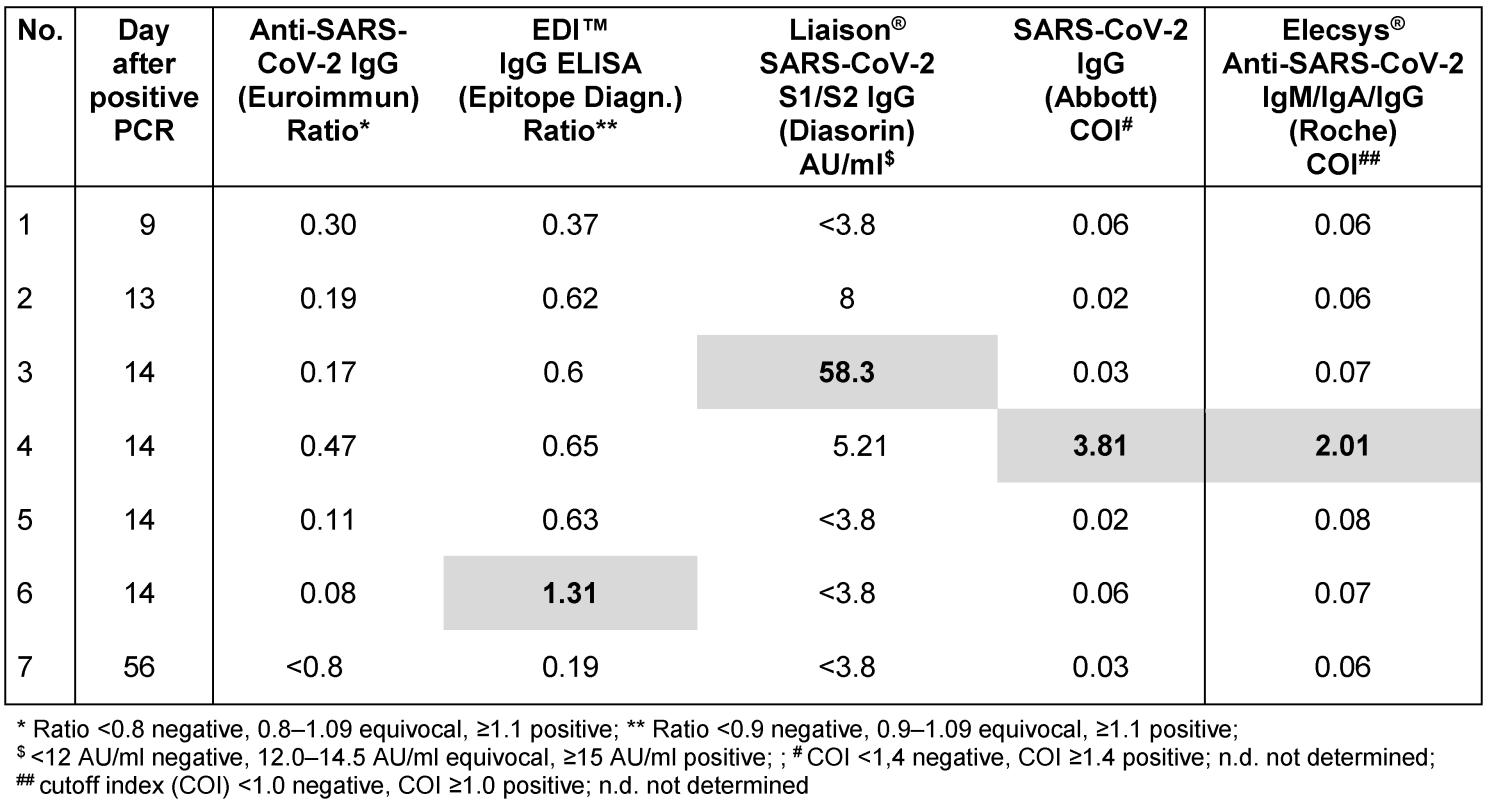
Sensitivity of five commercial SARS-CoV-2 assays in PCR-confirmed asymptomatic contact persons (n=7)

## References

[1] Kohmer N, Westhaus S, Rühl C, Ciesek S, Rabenau HF (2020). Brief clinical evaluation of six high-throughput SARS-CoV-2 IgG antibody assays. J Clin Virol.

[2] Lassaunière R, Frische A, Harboe ZB, Nielsen ACY, Fomsgaard A, Krogfelt KA, Jorgensen CS (2020). Evaluation of nine commercial SARS-CoV-2 immunoassays [Preprint]. medRxiv.

[3] Montesinos I, Gruson D, Kabamba B, Dahma H, Van den Wijngaert S, Reza S, Carbone V, Vandenberg O, Gulbis B, Wolff F, Rodriguez-Villalobos H (2020). Evaluation of two automated and three rapid lateral flow immunoassays for the detection of anti-SARS-CoV-2 antibodies. J Clin Virol.

[4] Tré-Hardy M, Wilmet A, Beukinga I, Dogné JM, Douxfils J, Blairon L (2020). Validation of a chemiluminescent assay for specific SARS-CoV-2 antibody. Clin Chem Lab Med.

[5] Infantino M, Damiani A, Gobbi FL, Grossi V, Lari B, Macchia D, Casprini P, Veneziani F, Villalta D, Bizzaro N, Cappelletti P, Fabris M, Quartuccio L, Benucci M, Manfredi M (2020). Serological Assays for SARS-CoV-2 Infectious Disease: Benefits, Limitations and Perspectives. Isr Med Assoc J.

[6] Burbelo PD, Riedo FX, Morishima C, Rawlings S, Smith D, Das S, Strich JR, Chertow DS, Davey RT, Cohen JI (2020). Detection of Nucleocapsid Antibody to SARS-CoV-2 is More Sensitive than Antibody to Spike Protein in COVID-19 Patients [Preprint]. medRxiv.

[7] Kellam P, Barclay W (2020). The dynamics of humoral immune responses following SARS-CoV-2 infection and the potential for reinfection. J Gen Virol.

[8] Lippi G, Salvagno GL, Pegoraro M, Militello V, Caloi C, Peretti A, Gaino S, Bassi A, Bovo C, Lo Cascio G (2020). Assessment of immune response to SARS-CoV-2 with fully automated MAGLUMI 2019-nCoV IgG and IgM chemiluminescence immunoassays. Clin Chem Lab Med.

[9] Long QX, Liu BZ, Deng HJ, Wu GC, Deng K, Chen YK, Liao P, Qiu JF, Lin Y, Cai XF, Wang DQ, Hu Y, Ren JH, Tang N, Xu YY, Yu LH, Mo Z, Gong F, Zhang XL, Tian WG, Hu L, Zhang XX, Xiang JL, Du HX, Liu HW, Lang CH, Luo XH, Wu SB, Cui XP, Zhou Z, Zhu MM, Wang J, Xue CJ, Li XF, Wang L, Li ZJ, Wang K, Niu CC, Yang QJ, Tang XJ, Zhang Y, Liu XM, Li JJ, Zhang DC, Zhang F, Liu P, Yuan J, Li Q, Hu JL, Chen J, Huang AL (2020). Antibody responses to SARS-CoV-2 in patients with COVID-19. Nat Med.

[10] Wölfel R, Corman VM, Guggemos W, Seilmaier M, Zange S, Müller MA, Niemeyer D, Jones TC, Vollmar P, Rothe C, Hoelscher M, Bleicker T, Brünink S, Schneider J, Ehmann R, Zwirglmaier K, Drosten C, Wendtner C (2020). Virological assessment of hospitalized patients with COVID-2019. Nature.

[11] Egger M, Bundschuh C, Wiesinger K, Gabriel C, Clodi M, Mueller T, Dieplinger B (2020). Comparison of the Elecsys® Anti-SARS-CoV-2 immunoassay with the EDI™ enzyme linked immunosorbent assays for the detection of SARS-CoV-2 antibodies in human plasma. Clin Chim Acta.

[12] Whitman JD, Hiatt J, Mowery CT, Shy BR, Yu R, Yamamoto TN, Rathore U, Goldgof GM, Whitty C, Woo JM, Gallman AE, Miller TE, Levine AG, Nguyen DN, Bapat SP, Balcerek J, Bylsma SA, Lyons AM, Li S, Wong AW, Gillis-Buck EM, Steinhart ZB, Lee Y, Apathy R, Lipke MJ, Smith JA, Zheng T, Boothby IC, Isaza E, Chan J, Acenas DD, Lee J, Macrae TA, Kyaw TS, Wu D, Ng DL, Gu W, York VA, Eskandarian HA, Callaway PC, Warrier L, Moreno ME, Levan J, Torres L, Farrington LA, Loudermilk R, Koshal K, Zorn KC, Garcia-Beltran WF, Yang D, Astudillo MG, Bernstein BE, Gelfand JA, Ryan ET, Charles RC, Iafrate AJ, Lennerz JK, Miller S, Chiu CY, Stramer SL, Wilson MR, Manglik A, Ye CJ, Krogan NJ, Anderson MS, Cyster JG, Ernst JD, Wu AHB, Lynch KL, Bern C, Hsu PD, Marson A (2020). Test performance evaluation of SARS-CoV-2 serological assays [Preprint]. medRxiv.

[13] Cervia C, Nilsson J, Zurbuchen Y, Valaperti A, Schreiner J, Wolfensberger A, Raeber ME, Adamo S, Emmenegger M, Hasler S, Bosshard PP, De Cecco E, Bächli E, Rudiger A, Stüssi-Helbling M, Huber LC, Zinkernagel AS, Schaer DJ, Aguzzi A, Held U, Probst-Müller E, Rampini SK, Boyman O (2020). Systemic and mucosal antibody secretion specific to SARS-CoV-2 during mild versus severe COVID-19 [Preprint]. bioRxiv.

[14] Zhao J, Yuan Q, Wang H, Liu W, Liao X, Su Y, Wang X, Yuan J, Li T, Li J, Qian S, Hong C, Wang F, Liu Y, Wang Z, He Q, Li Z, He B, Zhang T, Fu Y, Ge S, Liu L, Zhang J, Xia N, Zhang Z (2020). Antibody responses to SARS-CoV-2 in patients of novel coronavirus disease 2019. Clin Infect Dis.

[15] Jääskeläinen AJ, Kuivanen S, Kekäläinen E, Ahava MJ, Loginov R, Kallio-Kokko H, Vapalahti O, Jarva H, Kurkela S, Lappalainen M (2020). Performance of six SARS-CoV-2 immunoassays in comparison with microneutralisation. J Clin Virol.

[16] Wajnberg A, Mansour M, Leven E, Bouvier NM, Patel G, Firpo A, Mendu R, Jhang J, Arinsburg S, Gitman M, Houldsworth J, Baine I, Simon V, Aberg J, Krammer F, Reich D, Cordon-Cardo C (2020). Humoral immune response and prolonged PCR positivity in a cohort of 1343 SARS-CoV 2 patients in the New York City region [Preprint]. medRxiv.

[17] Meyer B, Torriani G, Yerly S, Mazza L, Calame A, Arm-Vernez I, Zimmer G, Agoritsas T, Stirnemann J, Spechbach H, Guessous I, Stringhini S, Pugin J, Roux-Lombard P, Fontao L, Siegrist CA, Eckerle I, Vuilleumier N, Kaiser L, Geneva Center for Emerging Viral Diseases (2020). Validation of a commercially available SARS-CoV-2 serological immunoassay. Clin Microbiol Infect.

[18] Liu Y, Yan LM, Wan L, Xiang TX, Le A, Liu JM, Peiris M, Poon LLM, Zhang W (2020). Viral dynamics in mild and severe cases of COVID-19. Lancet Infect Dis.

[19] Zhou F, Yu T, Du R, Fan G, Liu Y, Liu Z, Xiang J, Wang Y, Song B, Gu X, Guan L, Wei Y, Li H, Wu X, Xu J, Tu S, Zhang Y, Chen H, Cao B (2020). Clinical course and risk factors for mortality of adult inpatients with COVID-19 in Wuhan, China: a retrospective cohort study. Lancet.

